# A Lightweight API-Based Approach for Building Flexible Clinical NLP Systems

**DOI:** 10.1155/2019/3435609

**Published:** 2019-08-15

**Authors:** Zhengru Shen, Hugo van Krimpen, Marco Spruit

**Affiliations:** Department of Computing and Information Sciences, Utrecht University, Utrecht, Netherlands

## Abstract

Natural language processing (NLP) has become essential for secondary use of clinical data. Over the last two decades, many clinical NLP systems were developed in both academia and industry. However, nearly all existing systems are restricted to specific clinical settings mainly because they were developed for and tested with specific datasets, and they often fail to scale up. Therefore, using existing NLP systems for one's own clinical purposes requires substantial resources and long-term time commitments for customization and testing. Moreover, the maintenance is also troublesome and time-consuming. This research presents a lightweight approach for building clinical NLP systems with limited resources. Following the design science research approach, we propose a lightweight architecture which is designed to be composable, extensible, and configurable. It takes NLP as an external component which can be accessed independently and orchestrated in a pipeline via web APIs. To validate its feasibility, we developed a web-based prototype for clinical concept extraction with six well-known NLP APIs and evaluated it on three clinical datasets. In comparison with available benchmarks for the datasets, three high *F*1 scores (0.861, 0.724, and 0.805) were obtained from the evaluation. It also gained a low *F*1 score (0.373) on one of the tests, which probably is due to the small size of the test dataset. The development and evaluation of the prototype demonstrates that our approach has a great potential for building effective clinical NLP systems with limited resources.

## 1. Introduction

Today's technologies allow the accumulation of vast textual data, which consequently has boosted the popularity of NLP research. There has been a huge amount of papers published and a variety of NLP systems or toolkits crafted in multiple domains over the last two decades. Among them, clinical NLP occupies a large portion. There are clinical NLP systems, such as Apache cTAKES, that integrate different NLP tools to process clinical documents [1, 2]. There are also NLP tools which target certain specific clinical needs, including extracting medication information [3], identifying locations of pulmonary embolism from radiology reports [4], and categorizing pain status [5].

**Figure 1:**
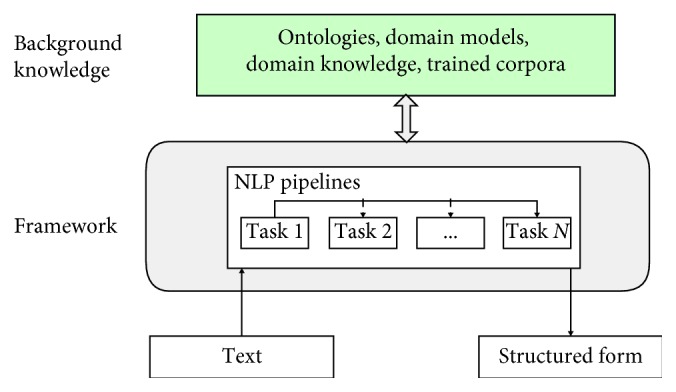
A general architecture of clinical NLP systems.

Figure 1 presents a general architecture of a clinical NLP system that contains two main components: background knowledge and framework [1]. Background knowledge contains ontologies, domain models, domain knowledge, and trained corpora. The widely used clinical domain knowledge is the Unified Medical Language System (UMLS) [6]. Framework refers to a software platform that integrates various NLP tasks or modules either sequentially or hierarchically into NLP pipelines. GATE and UIMA are the leading open-source frameworks [7, 8]. There are two levels of NLP tasks: low-level tasks and high-level tasks. Low-level tasks include tokenization, part of speech tagging, sentence boundary detection, and so on. High-level tasks refer to the semantic level processing such as named entity recognition, relation extraction, and sentiment analysis.

History has shown that building a successful clinical NLP system requires a tremendous amount of resources. For instance, it took a team from Columbia University 14 years to commercialize the MedLEE system [9]. The development of cTAKES started at the Mayo Clinic in 2006, and further external collaborations with four other universities in 2010 resulted in the first release of the current Apache project [2]. Therefore, creating reusable NLP pipelines based on open-source modular frameworks like GATE and UIMA becomes more reasonable [9, 10]. Although it dramatically reduces resources and level of expertise, we argue that it is not an efficient and effective solution for two main reasons. Firstly, nearly every NLP pipeline that is created to address a single specific clinical need, either rule or machine-learning based, has been proven to be useful for only its designated purposes [11]. Thus, reusability is difficult given the properties. Secondly, deploying cTAKES-based NLP pipelines implies a high cost of operation which requires installation and configuration of multiple components by NLP experts [12]. Besides, maintenance of a deployed NLP system requires a continuous investment.

With the purpose of simplifying and outsourcing the NLP implementation, software as a service, or SaaS, has been introduced to the NLP world during recent years [13]. SaaS generally refers to the mode of software delivery where end-users are charged with a monthly or annual subscription fee to utilize a set of functionalities over the Internet [14]. NLP systems distributed in the SaaS model are often available through web application programming interfaces (APIs) and named as NLP APIs or cloud-based NLP APIs [13, 15]. Many NLP APIs have emerged from both companies and universities and are growing popularly [7]. A few prominent examples are IBM Watson, Aylien, Lexalytics, and TextRazor [13]. From the cost-benefit perspective, these NLP APIs allow developers to rapidly create NLP-enabled tools without investing abundant resources on implementing necessary NLP techniques in codes and on regular maintenance. A number of applications based on NLP APIs were built [16–18].

To utilize NLP APIs, API-based frameworks have been produced [15,19–21]. API-based systems, also known as cloud-based, refer to tools that are built on external web APIs and having their functionalities partially or fully accomplished with one or a pipeline of APIs. Due to the growing popularity of web APIs in the software industry, API-based tools are abundant in companies. For instance, an API-based CMS (content management system) is utilized to save development resources and follow-up maintenance [22]. Furthermore, researchers have also investigated the approach in recent years. Rizzo and Troncy proposed the Named Entity Recognition and Disambiguation (NERD) framework that incorporates the result of ten different public APIs-based NLP extractors [21]. A web-based tool called TeXTracT was devised to support the setup and deployment of NLP techniques on demand [15]. Abdallah et al. developed a flexible and extensible framework for integrating named entity recognition (NER) web APIs and assessed it across multiple domains [19]. Although these tools exhibit promising results, few were built for clinical NLP or evaluated on clinical datasets. Therefore, it is safe to say that adopting these tools in clinical settings would be problematic due to the unique characteristics of the clinical domain. For example, privacy is considered to be of the utmost importance, but none of the above tools have taken it into consideration.

This paper thus proposes a lightweight framework which enables a rapid development of clinical NLP systems with external NLP APIs. The approach has the following advantages compared to traditional NLP frameworks: (1) fast development; (2) lower costs; (3) flexibility; and (4) programming language independent. The deployment is minimized by outsourcing both NLP tasks and background knowledge to external API services. Thus, NLP systems can be quickly and cost-efficiently developed based on the proposed framework. The framework is flexible in many aspects. To begin with, it supports the flexible combination of different NLP tasks from external APIs. Secondly, users have the freedom of choosing their preferred NLP API vendors, and multiple APIs can be integrated to achieve better results. To evaluate the framework, we have built a web-based open-source clinical NLP application.

## 2. Methods

### 2.1. Design Science

Our research followed the design science research as we built and evaluated the framework because of its strength and popularity in solving a real-world problem by designing and building an innovative IT artifact [23]. In our case, the artifact is a lightweight framework that facilitates clinical NLP systems development. We follow the design science research methodology (DSRM) proposed by Peffers et al., which consists of six steps: problem identification and motivation, definition of the objectives for a solution, design and development, demonstration, evaluation, and communication [24].

The DSRM is initiated by the (I) problem identification and motivation, which we addressed by literature study. Previous studies have described the general architecture of clinical NLP systems and how expensive it is to build them. Even though the introduction of modular NLP frameworks reduced the complexity of NLP systems, it is still challenging to create clinical NLP systems for many healthcare institutions due to limited resources. Based on the identified problem, we inferred the (II) objectives for a solution: creating a lightweight NLP framework that enables a rapid development of an API-based clinical NLP system. In the (III) design and development, we developed the framework based on the general architecture we identified, after which each of its components is explained in detail. To (IV) demonstrate and (V) evaluate the framework, a web-based open-source clinical NLP application was developed. Moreover, experiments were carried out with three clinical datasets to primarily examine whether external NLP APIs would deliver the state-of-the-art performance. The final step of the DSRM is the communication. The paper serves as the start of our (VI) communication on this topic.

### 2.2. Evaluation Design

Three English anonymized clinical datasets were used in our evaluation. Two of the datasets are obtained from the Informatics for Integrating Biology and the Bedside (i2b2) center: 2008 Obesity Challenge and 2009 Medication Challenge. The third dataset comes from a European clinical trial called OPERAM. Since the primary goal of our evaluation is to prove that external general-purpose NLP APIs can yield good performance on clinical data, we only used a subset of the two large i2b2 datasets.

- *2008 Obesity Challenge*. This dataset consists of 611 discharge letters. All discharge letters are annotated with 16 different medical condition terms in the context of obesity, including asthma, gastroesophageal disorder, and depression. Terms could be either annotated as being in the document, not being in the document, or undecided/unknown which was treated as a not being in the document. The strength of this dataset, concerning the aim of these tests, is that there are a lot of documents, snf its weakness is that it is only annotated for 16 abstract terms in the context of obesity. To simplify the experiment, we randomly selected 100 discharge letters and labeled each document with the medical conditions that are annotated as “present.”
- *2009 Medication Challenge*. 947 out of 1243 in total deidentified discharge letters have the gold standard annotations. Medication names in the annotations are used for the evaluation. By comparing the annotated medication names with those generated from our application, we calculate the evaluation metrics. We also randomly select 100 out of the 947 documents.
- *OPERAM Dataset*. The dataset consists of five discharge letters that have been used during the pilot of the OPERAM clinical trial [25]. Medical experts of the trial annotated these letters by both medical conditions and pharmaceutical drugs. Moreover, standardized clinical codes for each annotation are included. With this dataset, we aim to demonstrate the performance of our NLP application with clinical documents from practices, even though it is clear that the small size limits our findings.

We extracted entities of “medical condition” or “pharmaceutical drug” from the, in total, 205 clinical documents and then encoded them with UMLS. Based on the encodings, extracted entities were filtered so that distinct entities were extracted for each clinical document. In order to measure the performance of our extraction, we have used well-known metrics: precision, recall, and *F*1 score. They are computed from true positives (TP), false positives (FP), and false negatives (FN) for each document. As stated above, annotations of the 2008 Obesity Challenge are different from the other two datasets. To simplify the identification of positives and negatives, we divided annotations into two groups: positives that are in the text and negatives which are not mentioned. Therefore, comparing clinical entities extracted by our application to the ground truth, we calculate the following:

- TP: entities that were both extracted and annotated as positives
- FP: entities that were extracted as positives but were annotated as negatives
- FN: entities that were not extracted but were annotated as positives

Precision (1) represents the proportion of extracted positives that are annotated positives. On the contrary, recall (2) is the proportion of annotated positives that were correctly extracted as such. *F*1 score (3) is the harmonic mean of precision and recall:

(1) $$\begin{array}{c} \text{precision}=\frac{\text{TP}}{\text{TP}+\text{FP}}, \end{array}$$

(2) $$\begin{array}{c} \text{recall}=\frac{\text{TP}}{\text{TP}+\text{FN}}, \end{array}$$

(3) $$\begin{array}{c} F1\text{ score}=2\;*\;\frac{\text{precision }*\text{ recall}}{\text{precision}+\text{recall}}. \end{array}$$

## 3. Results

The section presents results in two parts: the framework and a web-based open-source clinical NLP application. The architecture lays down the technical groundwork, upon which the application was constructed. The following explains each of them in details.

### 3.1. A Lightweight NLP Architecture for Clinical NLP

**Figure 2:**
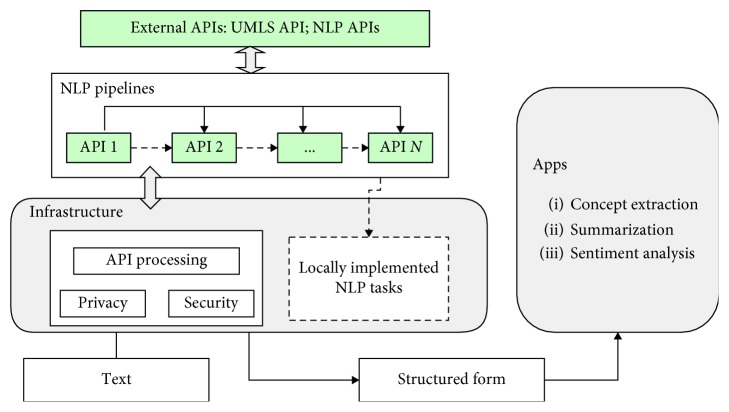
A lightweight NLP architecture for clinical NLP.

The architecture addresses the issues of existing clinical NLP applications, including interoperability, flexibility, and specific restrictions within the clinical field, such as privacy and security. The strength of our proposed architecture is shown in its capabilities: (1) freedom of assembling suitable NLP APIs either sequentially or hierarchically based on scenarios; (2) encoding clinical terms with comprehensive and standardized clinical codes; (3) the built-in deidentification function to anonymize clinical documents.  Figure 2 depicts its four main components: external APIs, infrastructure, NLP pipelines, and Apps.

#### 3.1.1. External APIs

In this architecture, two types of APIs, namely, an NLP API and a domain knowledge API, are included to parse unstructured medical text and map parsed terms against a medical metathesaurus, respectively. The NLP API provides various cloud-based NLP services that parse unstructured text for different purposes, including entity recognition and document classification. The domain knowledge API supports the mapping of medical text to concepts from the UMLS metathesaurus. As the most used biomedical database, UMLS contains millions of biomedical concept names and their relations. In addition, domain models and training corpora are available for specific clinical documents such as radiology reports, pathology reports, and discharge summaries [1]. The UMLS is a major part of the solution for standardization and interoperability as it maps terms extracted by multiple APIs to standardized codes such as ATC and ICD10.

#### 3.1.2. Infrastructure

The infrastructure layer prepares clinical data before sending them to external APIs by deidentification and adding authentications. Furthermore, it processes results received from external APIs for later integration. An optional component, locally implemented NLP techniques, is also incorporated.

- *API Processing*. The purposes of API processing are two-fold: (1) prepare clinical text before sending them to external APIs and (2) process results returned from external APIs. Given the difference between multiple APIs, data processing is inevitable to achieve interoperability. Specific API processing tasks include formatting clinical text for APIs requests, filtering results returned from APIs, and data conversion.
- *Privacy*. Privacy protection is a critical issue in clinical data sharing for both research and clinical practices, and privacy violations often incur legal problems with substantial consequences. The privacy component embedded in the infrastructure offers technical solutions to deidentify or anonymize patient-level data, such as CRATE [26] and DEDUCE [27]. CRATE is an open-source software system that anonymizes an electronic health records database to create a research database with anonymized patients' data. With CRATE implemented, our approach can directly use patients' data. In comparison with CRATE, DEDUCE is more lightweight. As a Python package, it processes sensitive patient information with commands like “*deduce*.*deidentify_annotations()*.”
- *Security*. The security component controls the access of clinical data and all external APIs. Authentication and encryption are added to safeguard data sharing via the Internet.
- *(Optional) Local NLP Tasks*. As discussed previously, an external NLP API grants no control of what NLP techniques to employ. In case some specific NLP techniques are required, our local NLP technique component provides a choice of implementing your own NLP techniques locally in a preferred language.

#### 3.1.3. NLP Pipelines

This layer provides a list of NLP services from which clinical applications can select the most suitable ones on demand. First of all, differences among NLP API providers in terms of their available NLP services are apparent. However, as shown in Table 1, there are also a number of common NLP services. Secondly, systematic studies have summarized some commonly used NLP techniques in clinical NLP applications [11]. By combining the common NLP services of various APIs and the useful NLP techniques in clinical settings, a shortlist of NLP services is selected for the architecture.

Moreover, multiple NLP services from different APIs can be integrated either sequentially or hierarchically for a single clinical NLP task. This enables clinical NLP applications to address the limitations of individual APIs caused by particular NLP techniques implemented and data employed to build it. More importantly, having a configurable NLP pipeline brings scalability and flexibility. For instance, a clinical concepts extraction enabled application can support combining entity extraction service from two or more of the NLP APIs in Table 1. However, interoperability between different NLP APIs becomes a challenge as both their inputs and outputs might vary considerably. Therefore, the NLP pipelines contain an integration component which facilitates the interoperability by implementing a proper integration strategy.

#### 3.1.4. Apps

In the application layer, clinical NLP-enabled applications for various needs can be created. They are produced either for performing a specific NLP task such as extracting diagnoses from discharge summaries and identifying drugs and dosage information from medical records or with a general purpose of processing unstructured clinical text. Existing NLP applications in clinical domains are categorized into the following groups:

- *Concept Extraction*. Kreimeyer et al. conducted a systematic literature review of NLP systems constructed to extract terms from clinical documents and map them to standardized clinical codes [11].
- *Text Classification*. Classification of free text in electronic health record (EHR) has surfaced as a popular topic in clinical NLP research. Koopman et al. devised a binary classifier to detect whether or not death is related to cancer using free texts of death certificates [28]. Other text classification examples in clinical settings cover classifying a complete patient record with respect to its eligibility for a clinical trial [29], categorizing ICU risk stratification from nursing notes [30], assessing inpatient violence risk using routinely collected clinical notes [31], and among others.
- *Sentiment Analysis*: Unlocking the subjective meaning of clinical text is particularly helpful in psychology. A shared task for sentiment analysis of suicide notes was carried out as an i2b2 challenge [32].

### 3.2. Prototype: API-Based Clinical Concept Extraction

To evaluate the architecture, a prototype that extracts clinical concepts from clinical free texts has been developed. This section first illustrates the design of its main components. Then, the prototype itself is presented.

#### 3.2.1. External NLP APIs

As described above, web NLP APIs have gained wide popularity over the last few years. Both academics and companies recognized the importance and extended their NLP systems with web APIs. As shown in Table 2, the prototype incorporates six leading NLP APIs from both academia and industry in its implementation. The selection is based on three criteria: (1) free or free trial available; (2) industrial APIs supported by big companies/teams; (3) academic APIs verified by peers.

#### 3.2.2. NLP Technique Implemented Locally

Studies have revealed that negation is very common in clinical reports [33, 34]. For instance, “no fracture,” “patient denies a headache,” and “he has no smoking history” often appear in clinical texts. In order to correctly extract clinical terms, negation detection becomes inevitable. However, given that most of the selected NLP APIs are tools for text processing and analysis in the general domain, the negation issue of clinical documents is not properly tackled, and they cannot filter out irrelevant information. Therefore, negation detection is implemented locally for the prototype. As the most well-known negation detection algorithm, NegEx has been adopted by a number of biomedical applications [35–37]. We implemented the algorithm to handle negation in this prototype.

#### 3.2.3. API Processing

NLP APIs first extract clinical terms which will be filtered by the local negator. Then the UMLS API transforms the filtered clinical terms to the standardized codes, such as ATC codes, ICD-10, or SNOMED, which ensures that the extracted clinical terms are interoperable after integration.

For each extracted term, the UMLS API returns its top 10 matched codes. These top matches are ranked on their similarity to the extracted term, with the first as the most similar one. The prototype captures the unique identifier of each matched code for later use.

As discussed above, when multiple APIs are applied for one task, results need to be integrated. The prototype employs a double weight system to integrate multiple APIs. The first weight system determines whether an extracted term is similar to another extracted term from the same document. The weight of a pair of two extracted terms is calculated based on their top 10 matches from the UMLS API and then is compared with the similarity threshold *γ*; if the weight is higher than the threshold, we consider it to be an equal term. The weight formula is shown as follows:

(4) $$\begin{array}{c} \left(\frac{\alpha }{4}\right)+\left(\frac{3\beta }{4}\right)≻\gamma , \end{array}$$

where *α* refers to the percentage of equal terms over all 10 terms and *β* is the percentage of equal terms over the top 3 terms. *α* and *β* are calculated based on the UMLS API matches of two extracted terms. The weight is a value between 0 and 1, 0 being that the terms are not similar at all and 1 being exactly the same. For a given NLP task, an initial value of *γ*=0.1 is recommended, and then according to the number of false positives and false negatives, we adjust the value of *γ* to achieve optimal output. The strategy of tuning these parameters is discussed further in Section 3.3.

The second weight system determines whether an extracted clinical term has enough cumulative weight from all NLP APIs. Since the performance of NLP APIs varies, a weight for each individual API is estimated by using the *F*1 scores calculated after testing each API on a small subset of clinical documents. The *F*1 score for each API is normalized to an extractor-weight *ω*. For each clinical term extracted, we sum the weights of the extractors the term was extracted by. If the weight is over the extractor threshold *θ*, it is considered to be actually extracted. If it is less, it is considered to be a false extraction. The weight is computed as follows:

(5) $$\begin{array}{c} \overset{n}{\underset{i=1}{\sum }}\omega_{i}, \end{array}$$

where *ω* is the weight of an NLP API and *n* refers to the number of API used. The pseudocode of the integration process is shown in Algorithm 1.

#### 3.2.4. Prototype

**Figure 3:**
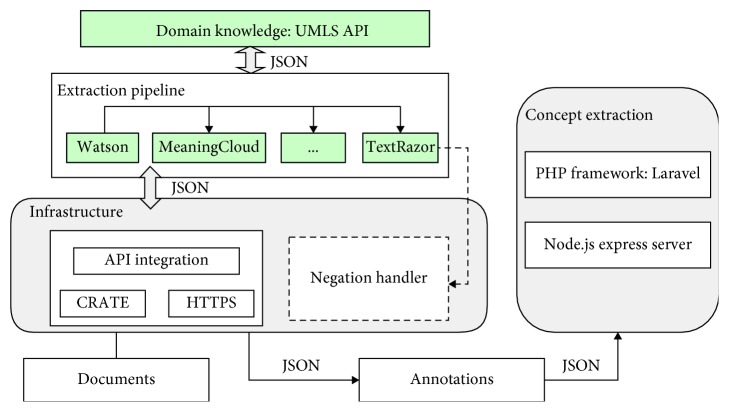
Prototype architecture.

**Figure 4:**
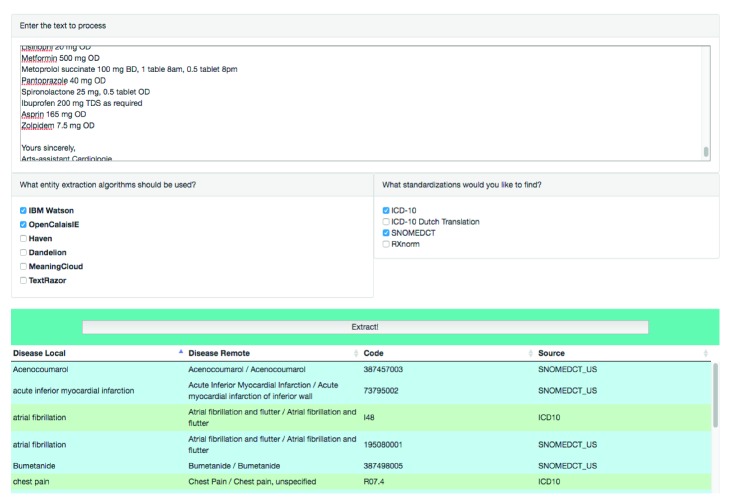
Prototype user interface of the multiple NLP API extraction pipeline. A demo video and source code are available online.

Figure 3 shows the overall functional components of the prototype, which is an instantiation of the proposed architecture. The prototype is a web application with a minimalistic user interface, developed with HTML5, CSS, JavaScript, and PHP for the back end. Given that many existing NLP APIs use JSON as the default format, JSON is the chosen format for data transferring between different components.  Figure 4 presents a screenshot of the application. Users need to provide clinical documents they want to process in the upper input field and then select APIs and coding standards. After clicking the Extract button, the results will be displayed in the table at the bottom. “Diseases Remote” lists the extractions of external NLP APIs, while “Diseases Local” represents results of combining external NLP APIs, the local negation handler, and the UMLS API. Unfortunately, the application is not accessible online due to a lack of API token management. Sharing our tokens online might incur a charge when there are a large number of API requests. Nevertheless, researchers are able to deploy their own version of the system with the source codes we share on GitHub at https://github.com/ianshan0915/MABNLP. A demo video is also available at https://youtu.be/dGk9NQGWYfI.

### 3.3. Evaluation Results

As explained before, the prototype comes with three hyperparameters that adjust the extraction outputs: negation (*κ*), term similarity threshold (*γ*), and extractor threshold (*θ*). The hyperparameter tuning was manually conducted by the researchers in the experiments.

The impacts of the controlling hyperparameters on the outputs of our experiments vary. First of all, negation surprisingly shows little positive influence as shown in Table 3. Its main reason probably lies in the fact that the implemented negation algorithm, NegEx, only uses negation cue words without considering the semantics of a sentence [34]. Implementation of more advanced algorithms, such as DEEPEN and ConText, will be conducted in future research. The higher *γ* value means a higher similarity threshold for entities to be merged, which results in a lower false positive and higher false negative numbers. By increasing the *θ* value, we want entities to be extracted by more APIs, and subsequently lower the number of false positives and increase the number of false negatives. However, higher values bring down the number of true positives. The aim is to strive for the best combination of these hyperparameters for each specific NLP task. The experiments suggested that the values of *γ* = 0.1 and *θ* = 0.35 are a decent starting point for further exploration.

Results have shown that the performance of the prototype is not consistent. Datasets like the obesity challenge can rely on our approach, but its reliability on datasets, such as the medication challenge and OPERAM dataset, need further improvement and evaluation.

Many NLP systems have been tested on the two i2b2 datasets, and there are benchmark performance metrics being published in the literature [38, 39]. We calculated the averages of top 5 best systems as the baselines. As displayed in Table 4, the prototype performs well and has great potential of being adopted for clinical concept extraction. In case of the OPERAM dataset, there is no benchmark. Therefore, its performance is evaluated from an expert intervention perspective. By comparing the automated extracted clinical concepts with the annotations, we estimate how well the prototype can be used to assist physicians during their manual extraction process. Unfortunately, feedbacks from physicians indicate that the prototype is not yet considered practically useful. Firstly, its poor performance in extracting medical conditions requires physicians to spend more time filtering out incorrect extractions. Secondly, the prototype fails to identify the associated dosages and frequencies of medications.

## 4. Discussion

We argue that outsourcing NLP tasks offers efficient NLP solutions for processing unstructured clinical documents. To begin with, outsourcing often leads to a reduction of both IT development and maintenance costs. Furthermore, a lower level of NLP expertise is required when external NLP services are used. A developer with limited knowledge of NLP could develop a clinical NLP application such as our prototype. Lastly, the architecture supports NLP services beyond clinical concept extraction. By adding a sentiment analysis NLP pipeline constructed by external NLP APIs, our prototype can perform sentiment analysis on clinical documents. For instance, changing from concept extraction to sentiment analysis can be accomplished by adjusting the API request parameters from “{“features”: “entities”}” to “{“features”: “sentiment”}.”

### 4.1. Evaluation Results

In comparison with the popular biomedical NLP component collections listed in [40], the main advantage of our proposed approach is its lightweight nature. The popular component collections, such as cTAKES, Bluima, and JCoRe, require an intensive IT resources investment including Java developers, NLP specialists with experience in the UIMA framework, and local hardware support. On the contrary, clinical institutions could start to process unstructured text with as little resources as possible due to the fact that our cloud-based approach outsources NLP to external NLP services. Moreover, Bluima has not been updated for four years. Instead of replacing the popular NLP tools, our approach should be considered as an alternative approach in the face of time and resource constraints.

### 4.2. Error Analysis

**Figure 5:**
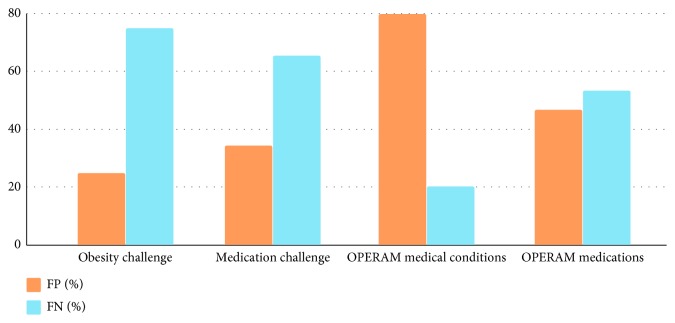
Error distribution of all the experiments, false positives vs false negatives.

An error analysis has been carried out in order to better understand the performance of the prototype. As explained in Section 2.2, there are two types of errors, namely, FPs and FNs.  Figure 5 shows the percentage of FP and FN errors in all experiments. First of all, one major source of errors in the two i2b2 datasets is false negatives, which means many annotated terms in the datasets are not extracted by our prototype. The high proportion of FNs is in great part attributed to the entity-type detection errors. Since some NLP APIs (MeaningCloud and Open Calais) are unable to extract pharmaceutical drug entities, it results in a lower amount of extracted entities and higher false negatives. Therefore, to enhance the performance, NLP APIs such as MeaningCloud and Open Calais might as well be excluded.

Nevertheless, the higher number of false positives led to an overall performance loss in the OPERAM medical conditions extraction. We found out that the problem lies in the annotation. For example, the sentence “Fall during the night, multiple hematomas. Orthostatic hypotension proven.” contains two medical conditions: hematoma and orthostatic hypotension. Hematoma was found by two out of six extractors; orthostatic hypotension was found by five out of six. However, neither of these two was annotated, most likely because the context of the sentence was in past tense and potentially not applicable to the current state of the patient.

### 4.3. Limitations and Future Research

There are a number of hurdles that prevent the adoption of our approach in daily practice. Further research is necessary to sufficiently address these concerns. First of all, practical implementation requires a more thorough privacy and security component. The privacy and security component is part of the proposed architecture and currently implemented in the prototype using CRATE [26] and HTTPS. However, since only anonymized datasets are used in the evaluation, the deidentification toolkit, CRATE, was not validated. Before the practical adoption, we need to first evaluate the performance of the privacy and security component with real-world clinical data.

Another concern lies in the computational efficiency of our approach, namely, execution time. As shown in the demo video, it takes about 20 seconds to process a discharge letter. In specific, the majority of time (15 seconds) goes to annotation in which extracted terms are first encoded with UMLS and then pairwise similarity between them is calculated. Since the prototype was running locally on a laptop with 8 GB RAM, we think it would become faster if we implement it on a larger server.

In practice, clinical NLP is employed to solve various clinical problems, ranging from entity extraction to cohort detection. Our research demonstrates that the proposed approach performs well on clinical concept extraction. It is crucial to conduct further evaluation on other tasks, such as cohort detection and sentiment analysis before adopting the approach in practice.

Last but not least, due to the wide adoption of health information systems (HIS) in healthcare institutions, developing a simple method that supports the integration of our approach with HIS would facilitate its implementation.

## 5. Conclusion

The proposed NLP architecture offers an efficient solution to develop tools that are capable of processing unstructured clinical data in the healthcare industry. With our approach, less time and resources are required to create and maintain NLP-enabled clinical tools given that all NLP tasks are outsourced. Moreover, the prototype built upon the approach produces satisfactory overall results, and its performance on certain datasets indicates that its practical application in clinical text processing, particularly clinical concept extraction, is promising. Nevertheless, high variance among different datasets brings concerns on its generalization and practicability.

## Figures and Tables

**Algorithm 1:**
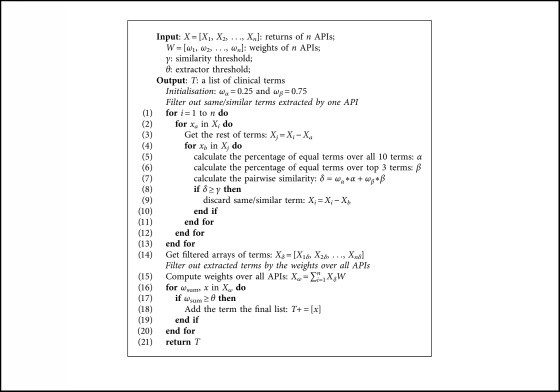
Pseudocode of the API integration algorithm.

**Table 1:** NLP services of common NLP API providers.

NLP API	Available NLP services
IBM Watson NLU	Entity extraction, concept extraction, relation extraction, text classification, language detection, and sentiment analysis
Aylien	Article extraction, entity extraction, concept extraction, summarization, text classification, language detection, semantic labeling, sentiment analysis, hashtag suggestion, image tagging, and microformat extraction
Lexalytics	Sentiment analysis, concept extraction, categorization, named entity extraction, theme extraction, and summarization
Meaning Cloud	Topic extraction, text classification, sentiment analysis, language detection, and linguistic analysis (POS tagging, parsing, and lemmatization)
Alchemy API	Entity extraction, concept tagging, keywords extraction, relation extraction, text classification, language detection, sentiment analysis, microformat extraction, feed detection, and linked data
TextRazor	Entity extraction, disambiguation, linking, keywords extraction, topic tagging, and classification
Developer Cloud	Concept extraction, translation, personality insights, and classification
Open Calais	Entity extraction, relation extraction, and sentiment analysis
Dandelion API	Entity extraction, text classification, language detection, sentiment analysis, and text similarity
Haven OnDemand	Autocomplete, concept extraction, document categorization, entity extraction, language detection, sentiment analysis, and text tokenization

**Table 2:** NLP APIs selected for the prototype.

API	Fee	Company/team	References
IBM Watson NLU	Free trial	IBM	https://www.ibm.com/watson/developercloud/natural-language-understanding/api/v1/
MeaningCloud	Free trial	MeaningCloud LLC	https://www.meaningcloud.com/developer/documentation
Open Calais	Free trial	Thomson Reuters	http://www.opencalais.com/opencalais-api/
Haven OnDemand	Free trial	Hewlett Packard	https://dev.havenondemand.com/apis
TextRazor	Free trial	TextRazor Ltd.	https://www.textrazor.com/docs/rest
Dandelion API	Free trial	Spaziodati	https://dandelion.eu/docs/

**Table 3:** Impact of negation from the experiments.

Dataset	Negation (*κ*)	Recall	Precision	*F*1 score
Obesity challenge	True	0.733	0.939	0.823
	False	0.805	0.925	0.861

Medication challenge	True	0.62	0.835	0.712
	False	0.636	0.838	0.724

OPERAM medical conditions	True	0.594	0.271	0.373
	False	0.594	0.271	0.373

OPERAM medications	True	0.795	0.816	0.805
	False	0.795	0.816	0.805

**Table 4:** Overall results on three datasets.

Dataset	*κ*	*γ*	*θ*	Recall	Precision	*F*1 score
Obesity challenge	False	0.1	0.2	0.805	0.925	0.861
		Baseline^*∗*^		**0.771**	**0.815**	**0.787**
Medication challenge	False	0.1	0.35	0.636	0.838	0.724
		Baseline^*∗*^		**0.794**	**0.845**	**0.818**
OPERAM medical conditions	True	0.1	0.5	0.594	0.271	0.373
OPERAM medications	False	0	0.35	0.795	0.816	0.805

^*∗*^Average of the top 5 best systems from the challenge.

## Data Availability

Source code and the OPERAM dataset are available at the GitHub repository https://github.com/ianshan0915/MABNLP. The two i2b2 datasets are accessible from https://www.i2b2.org/NLP/DataSets/Main.php. Finally, a demo video of the prototype is available at https://youtu.be/dGk9NQGWYfI.
